# Corrosion Behavior of Incoloy^®^800H, Hastelloy^®^G35^®^ and 316L Stainless Steel in the Molten Eutectic Fluoride Mixture FLiNaK and Its Vapors

**DOI:** 10.3390/ma16072679

**Published:** 2023-03-28

**Authors:** Ambati Ramu, Viliam Pavlik, Veronika Sillikova, Miroslav Boca

**Affiliations:** Institute of Inorganic Chemistry, Slovak Academy of Sciences, Dubravska cesta 9, 854 36 Bratislava, Slovakia

**Keywords:** FLiNaK salt, Incoloy^®^800H, vapors, Hastelloy^®^G35^®^, 316L stainless steel

## Abstract

This paper discusses the findings of a corrosion experiment which investigates a transition area which is between the lower area and upper area of the sample. In this experiment, the lower half of the sample surface is exposed to molten salt and the upper half of the sample surface is exposed to the vapors generated by molten FLiNak salt. Incoloy®800H and Hastelloy®G35® alloys and 316L stainless steel were selected for the corrosion experiment on the basis of their Cr content. The experiment was conducted at 600 °C for a period of 100 h. The results of the experiment show that, in the transition area, no abrupt change in corrosion mechanism takes place; the experiments also give us information on how the degree of degradation varies across the three areas of the samples. The experiment also showed two distinct corrosion mechanisms operating in the test samples: intergranular corrosion in the SS316L stainless steel test sample and continuous corrosion in Hastelloy®G35®. The results also show a progressive reduction in the concentrations of F, K and Na in the upper areas compared to the lower areas for Hastelloy©G35®. Cr is shown to have a critical role in the corrosion process, even when the sample surface is not in direct contact with the molten salt and is only in contact with the vapors generated by the molten salt.

## 1. Introduction

Molten salt reactors (MRSs) have attracted interest across the world in recent years due to their better safety profile when compared to water-cooled reactors [1,2,3,4]. The defining feature of MSRs is the use of molten salts, rather than water, as a heat transfer medium, which makes them safer. In terms of selection of the salts to be used in MRSs, fluorides, chlorides and nitrides have been studied [1,5]. However, on the basis of its advantageous thermophysical properties, such as small neutron absorption cross section and low melting point (among others), the eutectic salt mixture FLiNaK (LiF-NaF-KF: 46.5–11.5–42.0 mol.%) was found to be best suited for these applications [1,2,5,6,7].

However, at high operating temperatures, such as in MSRs, the molten FLiNaK salt is corrosive in nature towards structural materials, as described in the articles [8,9,10,11,12,13,14]. Detailed research into the mechanisms behind the corrosion phenomenon has revealed that corrosion is initiated by the presence of impurities in the FLiNaK salt: for example, H_2_O forms HF—Equation (1)—and then the HF reacts with other impurities and alloying elements such as Cr, Fe, Ni and others to form fluorides, shown for Cr and Fe in Equations (2) and (3). These fluorides, CrF_2_, CrF_3_, FeF_2_, FeF_3_, NiF_2_ and others, dissolve in the molten salt, thereby exposing the structural material to further corrosion. This process is thoroughly explained in articles [15,16,17,18,19,20].

H_2_O + 2F^−^ ⇔ O_2_ + 2HF (1)

Cr + xHF ⇔ (x/2)H_2_ + CrFx (2)

Fe + xHF ⇔ (x/2)H_2_ + FeFx (3)

In addition to the corrosion taking place in the molten salt environment as a result of the impurities that are present, volatility and off-gases generated by the FLiNaK salt also cause corrosion of the structural material. However, there are only limited data regarding this process [21,22,23,24,25].

To understand the corrosion caused by volatility and off-gases from FLiNaK salt, preliminary work was carried out on Ni-WC cemented carbide [26]. In that work, one sample was submerged into the molten salt, to simulate the corrosion taking place in the molten salt, and another sample was suspended above the molten salt to simulate the corrosion taking place due to the volatility and off-gases generated by the molten FLiNaK salt. This allowed the results of the two areas (molten salt areas and vapors areas) to be obtained independently of each other.

However, in salt-containing vessels, the corrosion mechanism does not change abruptly when going from a molten salt environment to a vaporous environment. There may be a transition area where both corrosion mechanisms are be active and could affect the material. We propose to define the transition area as starting from the end-point of the molten area, but there is no sharp boundary between the transition zone and the vapor zone (this will change with temperature and will likely also be different for different alloys).

To obtain data from all three areas (molten salt area, transition area, and volatility and off-gases area), this experiment submerged the sample halfway into the molten salt area, exposing the other half to the vapor, thereby creating the required three areas (molten salt area, transition area and vapor-exposed area) in one sample. By doing so, the sample will provide information on corrosion mechanisms in (i) the molten salt environment, (ii) the area in close proximity to the molten salt (transition area) and (iii) the area where volatility and off-gases generated by the FLiNaK salt come into contact with the surface of the sample.

## 2. Materials and Methods

The Incoloy^®^800H and Hastelloy^®^G35^®^ alloys and 316L stainless steel were selected for this experiment on the basis of their Cr content, as Cr is more susceptible to corrosion, as shown in refs. [19,27]. Incoloy^®^800H has the advantage of being certified for use up to 700 °C [28]. The composition of the selected alloys is given in Table 1. Incoloy^®^800H and SS316L were procured from Bibus metals s.r.o (CZ) and Hastelloy-G35 was obtained from Haynes international (CH).

Due to the different suppliers of the materials, it was not possible to obtain uniform dimensions of the samples across all three alloys, so it should be borne in mind that the sample sizes in our experiments varied with the material. However, different samples of the same alloy were cut to the same dimensions, which were as follows: for SS316L stainless steel, 5 mm × 5 mm × 15 mm; for Hastelloy©-G35, 5.2 mm × 4 mm × 17 mm; and finally, for Incoloy©-800H, 8 mm × 4 mm × 12 mm.

After cutting the samples to the dimensions specified, the samples were then mechanically polished using a Struers LABOSYSTEM machine. Progressively finer grades of SiC paper were used, up to 4000 grids, in order to obtain a mirror-like finish on the surface. The polished samples were then ultrasonically cleaned in acetone, to remove any residue from the polishing process.

**Figure 1:**
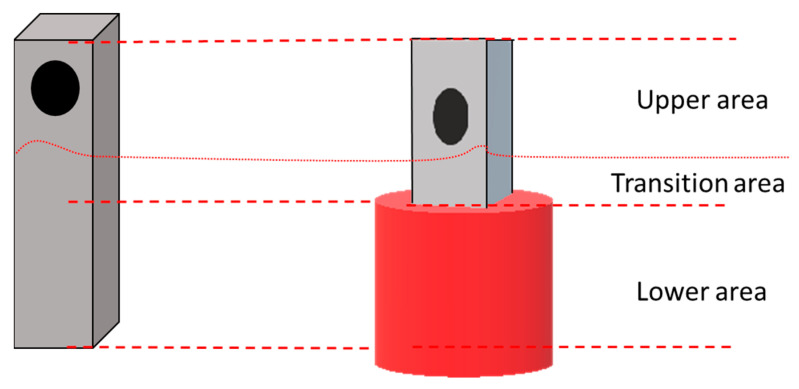
The image shows the three area of the sampe which are invstigated.

The initial weights of the polished samples were measured to five decimal places using a high-precision balance. After weighing, FLiNaK salt for the corrosion experiment was prepared from high purity (99%) LiF, NaF and KF as described in ref. [29]. Next, the prepared salt was placed into the glassy carbon crucible, after which the samples were immersed halfway into the salt as shown in Figure 1. Finally, the crucible opening was closed with a carbon lid and placed into the furnace.

**Figure 2:**
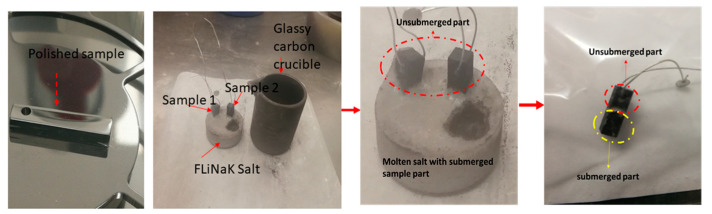
Sequence of processes taking place in the corrosion experiment. The two images on the left show the polished sample and the experimental procedure, and the second two images show the post-experiment process.

The crucible and its contents were then heated to 600 °C for a period of 100 h in an argon atmosphere. After the stipulated time of 100 h, the furnace was turned off and allowed to cool spontaneously to room temperature. Finally, after the completion of the experiment, the samples were separated from the solid salt, as shown in Figure 2.

The salt-covered test samples were cleaned with 1.0 M Al(NO_3_)_3_ solution, to remove the salt and reveal the underlying surface, as described in [15]. The cleaned samples were then weighed in order to calculate the weight change after corrosion. For morphology, elemental mapping and study of the corrosion mechanism, the samples were individually examined with a Zeiss EVO 40HV electron microscope (Carl Zeiss). For the elemental distribution in the sample, an EDX (Energy-dispersive X-ray spectroscopy) analyzer unit Röntex-Quantax BRUKER AXS, with WD distance 12.9 mm and HV 10 kV, was used. Following the surface analysis, the samples were cut along their whole length for the cross section analysis, the results of which are described in the Results section. After the experiment, the FLiNaK salt was separated from the sample and crushed. It was then analyzed with XRD to determine the presence of corrosion products dissolved in the salt. For the X-ray analysis, a PANalytical diffractometer with (Cu kα radiation with Ni β-filter) was used. The 2θ was 5–90° and steps 0.13; the measuring steps were 46.665.

## 3. Results

### 3.1. Weight Loss

Mass change data from the literature show different patterns according to the type of exposure to the molten salt: samples which were completely submerged in the molten salt environment showed mass loss, as in articles [17,30]. In contrast, samples which were not submerged in the molten salt, but only exposed to volatiles and off-gases from the molten FLiNaK salt, as in refs. [21,31], showed mass gain. However, in this experiment, where one half of each of the three test samples was immersed into the salt (submerged sample part) and the other half only exposed to the vapors (unsubmerged part) (see Figure 2), the sample shows overall mass loss.

**Figure 3:**
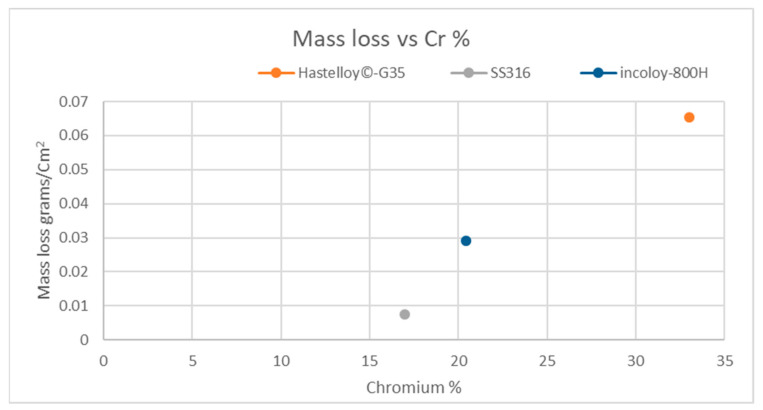
Graph showing that the mass loss in grams/cm^2^ increases with Cr content in the samples.

In this experiment, the mass loss of the three test samples shows a correlation between Cr content in the test samples and the mass loss; Hastelloy^®^-G35^®^ has the largest mass loss, followed by Incoloy^®^-800H and finally SS316L stainless steel, which shows the lowest mass loss. Thus, the higher the content of Cr in the alloy, the higher the mass loss (Figure 3).

### 3.2. X-ray Data

The XRD (X-ray powder diffraction) data of the salts from the three experiments showed the presence of LiF, KF and NaF (Supplementary Materials Figure S1); other corrosion products may also have dissolved into the molten FLiNaK salt but remained unidentified, due to the dissolved quantity being lower than the detection limit of the XRD machine.

### 3.3. Surface Morphology

**Figure 4:**
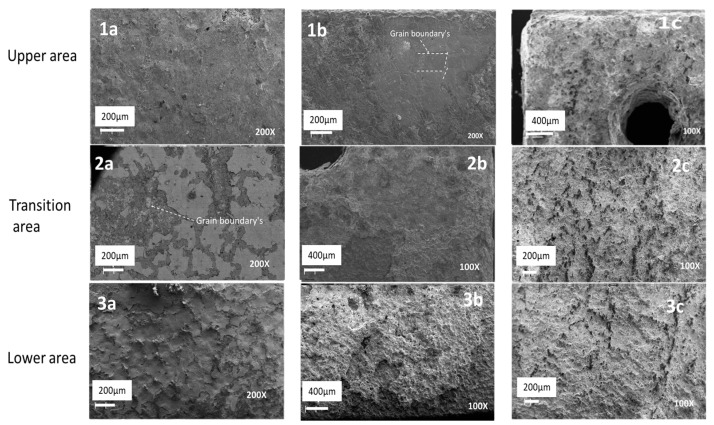
The surfaces of the three areas of SS316L stainless steel (**1a**–**3a**), Incoloy^©^-800H (**1b**–**3b**) and Hastelloy^®^G35^®^ (**1c**–**3c**) are shown in the image.

Images of the surface morphology are shown in Figure 4. Starting from the upper left, Figure 4 image 1a shows (at 200× magnification) the upper area of the SS316L sample, which was exposed to the high-temperature volatiles and off-gases from the molten salt. The upper area has a smooth surface and does not show much degradation from the corrosion process. Below the “upper area” is the “transition area” (Figure 4, image 2a at 200× magnification), which was in close proximity to the molten salt (or, in some cases, may have come into contact with the molten salt during the experiment). The transition area shows a grain boundary marked with a white arrow (Figure 4, image 2a at 200× magnification) and corrosion scale covering part of the surface. The lower area, which was in contact with the molten salt, shows a high degree of degradation. The surface of the lower area is covered with a corrosion layer (See Figure 4 image 3a).

The upper area of the Incoloy^©^-800H sample shows grain boundaries and a smooth surface indicated with white dashed lines (Figure 4 image 1b at 200× magnification). The smooth surface is similar to the upper area of SS316L. The transition area in closer proximity to the molten salt has high degradation (Figure 4 image 2b at 100× magnification). The lower area, where molten salt has come into contact with Incoloy^®^-800H, is also highly degraded (Figure 4, 3b at 100× magnification) and partially covered by a corrosion layer.

The Hastelloy^®^-G35^®^ sample shows a high degree of degradation in all three areas, regardless of whether or not they have come in contact with the molten salt (Figure 4 images 1c, 2c, and 3c).

### 3.4. EDX Elemental Mapping

It should be noted that, in all the reported elemental mapping results, the lower areas are taken as a reference with which to compare the two other areas (the transition areas and the upper areas), as the lower areas are in direct contact with the molten salt, allowing the full corrosion process to take place. This approach has been adopted because we are interested in how the two other areas differ from the lower area in terms of the extent and mechanism of corrosion.

#### 3.4.1. Elemental Mapping of SS316L Stainless Steel

**Figure 5:**
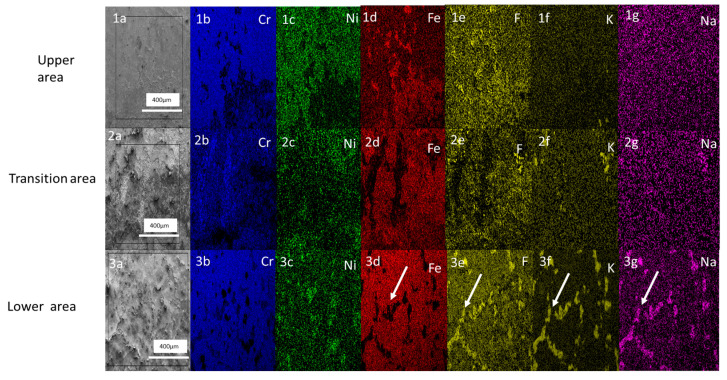
Surface elemental mapping of the SS316L test sample’s three areas. Images (**1a**–**1g**) show the elemental distribution of the upper area of the sample. Images (**2a**–**2g**) show the elemental distribution of the transition area. Images (**3a**–**3g**) show the elemental distribution of the lower area. The white arrows in the images 3d–3g show the grain boundary.

The elemental mapping of the lower area of the SS316L stainless steel test sample (see image 3a–3g, Figure 5) shows a high concentration of F, K and Na together (see image 3e–3g, Figure 5), which is marked with a white arrow in each image. In contrast, there is no visible Fe and Cr concentration in those places where F, K and Na are present in the EDX mapping of the lower area (see images 3b–3d, Figure 5). The areas of high deposition of K, F and Na can be assumed to be grain boundaries and the corrosion mechanisms can thus be assumed to be intragranular corrosion. This concurs with previous findings of corrosion at grain boundaries [32].

Comparing the findings of the elemental mapping in the transition area (see image 2a–2g, Figure 5) with the elemental mapping in the lower area, it can be observed that grain boundaries were not visible in the transition area and that a high concentration of F, K and Na is also absent along the grain boundary, unlike in the lower area of the sample surface. The comparison of the upper area with the lower area also shows no indication of grain boundaries (see image 1a–1g, Figure 5).

#### 3.4.2. Cross Section of SS316L Stainless Steel

**Figure 6:**
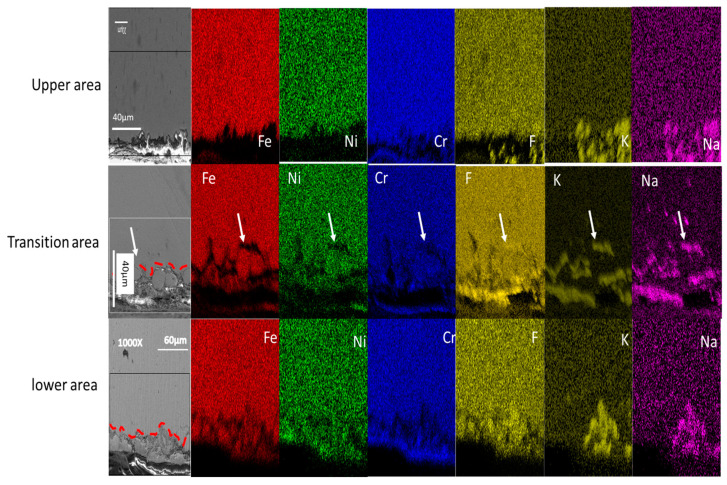
Cross section of SS316L stainless steel with the depth of penetration of the elements into the base metal.

The lower area is taken as a reference with which to compare the two other areas (the transition area and the upper area), as in the previous section. In the lower area, it is clearly visible that grain boundaries have been most strongly affected and that intergranular corrosion is driving the corrosion in SS316L stainless steel. The EDX mapping of the cross section shows that F, K and Na had diffused along the grain boundaries of the SS316L stainless steel sample, as shown by the white arrows (Figure 6). The EDX mapping also shows low concentration of Cr, Ni and Fe along the grain boundary, also shown by arrows in Figure 6.

In the transition area, grain boundaries with intergranular corrosion were also visible, as seen in the areas indicated with white arrows close to the surface. F, K and Na also penetrated into the metal matrix in a similar way as in the lower area.

In the upper area, the depth of corrosion was very shallow and it was hard to determine the corrosion mechanism, but F, K and Na had penetrated into the metal matrix in this case as well, as shown in the first row of Figure 6.

#### 3.4.3. Surface Mapping of Incoloy^®^-800H

**Figure 7:**
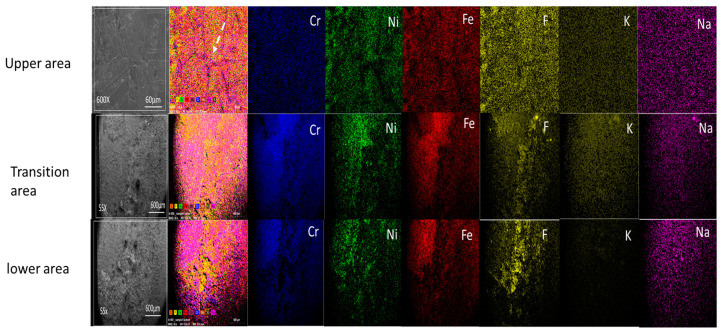
Surface mapping of Incoloy^®^-800H. The image also shows grain boundary in the upper area, as indicated by the white arrow.

The elemental mapping of the lower area of the Incoloy^®^-8000H (see Figure 7) does not reveal any grain boundaries or clearly reflect any corrosion mechanism, unlike the SS316L stainless steel lower area (see image 3a–3g, Figure 5); this may be because the surface is covered by a corrosion layer. The transition area does not reveal any grain boundaries or corrosion mechanism either. However, in the upper area, there are grains and grain boundaries visible, as indicated with white arrows (top row of Figure 7), indicating that intergranular corrosion is the corrosion mechanism in the upper area for Incoloy^®^-800H.

#### 3.4.4. Cross Section of Incoloy^®^-800H

**Figure 8:**
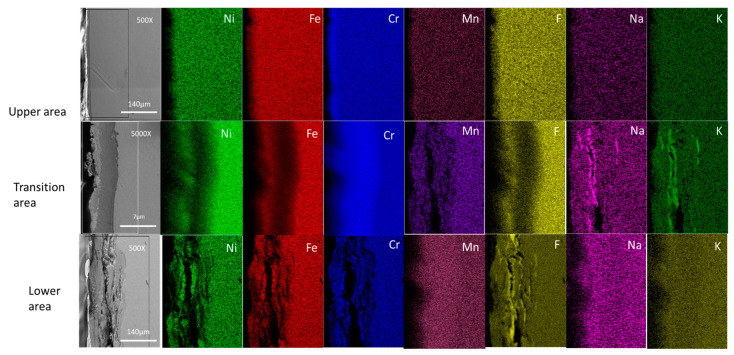
Cross section of Incoloy^®^800H.

The mapping of the cross section did not show any indications of the corrosion mechanism. However, the lower area has a high degree of degradation, with diffusion of F, Na and K into the metal, as shown in Figure 8.

#### 3.4.5. Mapping the Surface of the Hastelloy^®^G35^®^ Sample

**Figure 9:**
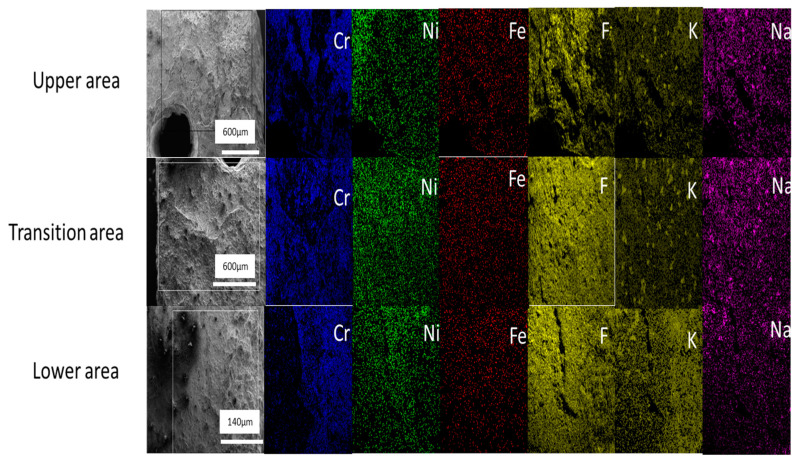
Surface mapping of Hastelloy^®^G35^®^ with concentration of different elements on the surface.

Surface mapping of Hastelloy^®^G35^®^ did not reveal any preferential corrosion mechanics in any of the three areas of the test sample surface (Figure 9), as was seen in SS316L stainless steel and, to a slight extent, in Incoloy^®^-800H.

#### 3.4.6. Cross Section Mapping of Hastelloy^®^G35^®^

**Figure 10:**
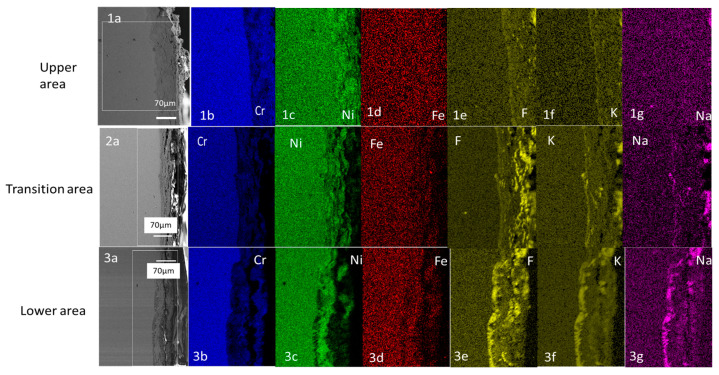
Cross-sectional elemental mapping of Hastelloy^®^G35^®^is shows. Images (**1a**–**1g**) show the elemental distribution of the upper area. Images (**2a**) show the and the consecutive images to its right show shows the elemental distribution of the transition area. Images (**3a–3g)** show the elemental distribution of the lower area.

Cross-sectional mapping of the Hastelloy^®^G35^®^ does not show intergranular corrosion in any of the three areas (Figure 10). Starting with the lower area, it is clear that there was a continuous inward diffusion of F, K and Na into the metal matrix, up to a depth of 70 µm. The outward diffusion of Cr and Fe remained constant.

Next, the transition area shows an inward diffusion of F, K and Na, but the concentration was lower than the concentration of F, K and Na in the lower areas. However, the depth of corrosion remained the same. The outward diffusion of Cr, Fe and Ni remained the same as in the lower area.

Finally, in the upper area, the F, K and Na inward diffusion was low when compared to the transition and lower areas; this is because the amounts of F, K and Na encountered by the upper area (which was only in contact with the vapors) are much lower than the amounts encountered by the areas in contact with bulk FLiNaK. Therefore, penetration of the sample was far lower. The outward diffusion of Cr, Fe and Ni was slightly higher than the inward diffusion of F, K and Na in the upper area.

## 4. Discussion

Immersion of the test sample halfway into the molten salt bath, leaving it semi-exposed to vapors at high temperatures, revealed that higher Cr content in the alloy leads to higher mass loss of the alloy (see Figure 3). This finding is consistent with the data of other research, in which Ni-based alloys with different Cr contents were tested for corrosion resistance in molten FLiNaK salt [33,34]. It suggests that mass loss is dependent on the Cr content of the alloy rather than on the corrosion environment.

The main reason that Cr content so strongly determines the corrosion response is that Cr reacts with dissolved fluorides of Ni and Fe because Ni and Fe fluorides are thermodynamically less stable, as described in Equations (4)–(7), whereas Cr forms the most stable fluoride. The formation of such fluorides has been explained well [8,27,35]; however, for completeness, simplified possible corrosion reactions are given below, together with their free energies:

Fe + 2FeF_3_ → 3FeF_2_   −33.78 kcal/mol (4)

Fe + NiF_2_ → FeF_2_ + Ni   −16.168 kcal/mol (5)

Cr + FeF_2_ → CrF_2_ + Fe   −19.121 kcal/mol (6)

Cr + NiF_2_ → CrF_2_ + Ni   −35.289 kcal/mol (7)

Analyzing the surface morphology of the test samples, it is clear that the lower zones of the three test samples are highly degraded. The degradation is clearly caused by the interaction of the molten salt and the sample surfaces (see Figure 5). In the case of the lower areas of the SS316L, the degradation clearly showed an intergranular corrosion mechanism to be operating (see Figure 6), which is consistent with the finding of ref. [30].

When comparing the transition area with the lower area, only small differences can be observed for both the SS316L stainless steel and Incoloy^©^-800H test samples. In the transition area, a corrosion layer was observed in both the SS316L stainless steel and Incoloy©-800H test samples and, to some extent in the SS316L stainless steel, grain boundaries were exposed (see Figure 6 and Figure 7).

The transition area of Hastelloy^®^G35^®^ had high surface degradation, similar its lower area of the Hastelloy^®^G35^®^. This indicates that whether a high degree of degradation of the transition area surface is seen is also dependent on the alloying elements present: in Hastelloy^®^G35^®^, the high Cr content leads to significant diffusion of Cr out to the surface, forming fluorides as described in refs. [8,27,35].

In the upper areas, a clear, smooth surface for SS316L and grain boundaries for Incoloy©-800H were visible. This confirms that, if the material does not come into direct contact with the molten salt, both the degradation and the corrosion will be lower. It also showed that, in Incoloy©-800H, intergranular corrosion was operating.

However, the high degradation for Hastelloy^®^G35^®^, as seen in the lower and transition areas, continued even into the upper area. This strengthens the argument that Cr is the main driving force behind the degradation and corrosion, as it reacts vigorously with the high temperature vapors from the molten salt, leading to a higher degree of corrosion when Cr content is higher.

**Figure 11:**
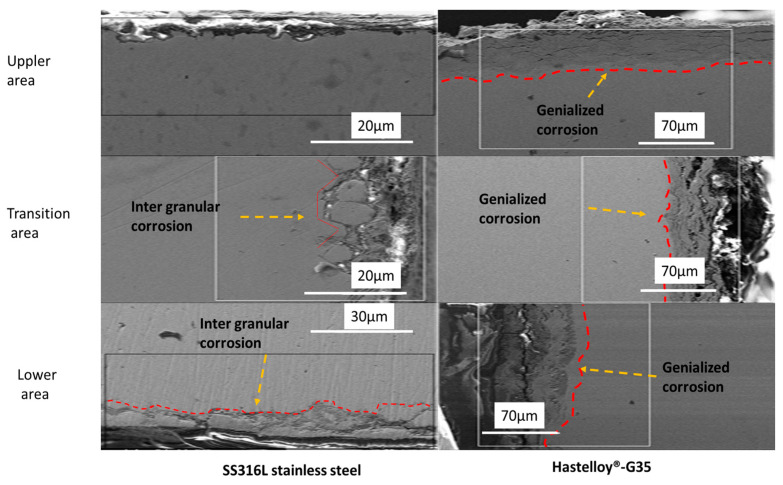
Cross sections of SS316L stainless steel and Hastelloy^®^G35^®^. The SS316L sample shows intergranular corrosion. In the Hastelloy^®^G35^®^ sample, general corrosion can be seen.

When compared, the SS316L stainless steel and Hastelloy^®^G35^®^ test sample cross sections show two distinct corrosion mechanisms operating in the FLiNaK salt environment (See Figure 11). In SS316L, intergranular corrosion was found to be the operating mechanism for corrosion. On the other hand, Hastelloy^®^G35^®^ did not have any specific dominant mechanism such as that seen in the SS316L test sample. In Incoloy-800H, the data from other research also show intergranular corrosion [34], the same as for the SS316L test samples.

**Figure 12:**
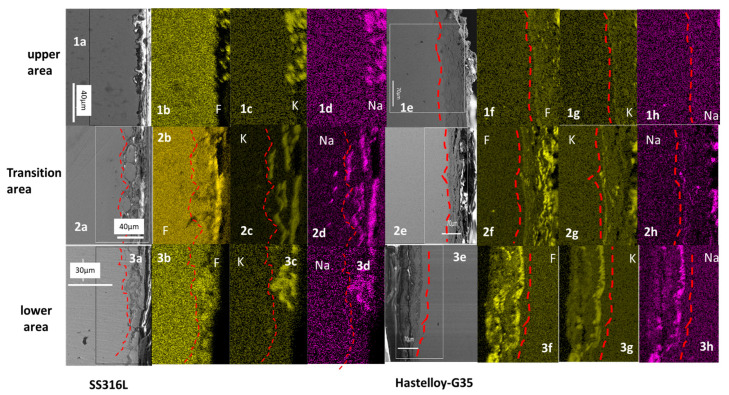
Cross-sectional mapping of the upper area (**1a**–**1d**), transition are (**2a**–**2d**) and lower (**3a**–**3d**) of SS316L.the upper area of (**1e**–**1h**), transition area of (**2e**–**2h**) and lower are of (**3e**–**3h**) ofHastelloy^®^G35^®^ areas area shown.

EDX mapping of the SS316L cross section shows inward diffusion of F, K and Na (see Figure 12) into the metal matrix. The thickness of the corrosion layer in the lower and transition areas was around 20 μm, using the scale in Figure 12 for measurement. However, the depth of corrosion in the upper area was shallow and hard to measure (see Figure 11), which confirms that, if the alloy does not come into contact with the molten salt or it is far away from it, the corrosion will be shallow.

In contrast to the SS316L cross section, in the Hastelloy^®^G35^®^ cross section the inward diffusion of F, K and Na was different in each area. In the lower area, the inward diffusion was high, as can be seen in Figure 12, images 3e–3h. As we move to the transition area, the inward diffusion of F, K and Na becomes smaller. This indicates that, in the case of Hastelloy^®^G35^®^, the further away the surface from the molten salt, the lower the corrosion when compared to the lower areas. The upper areas show even lower inward diffusion of F, K and Na, due to corrosion from vapors being dominant there.

## 5. Conclusions

- The experiment showed a single corrosion mechanism operating in each sample. Stainless steel 316L has intergranular corrosion and Hastelloy^®^G35^®^ has generalized corrosion. However, the mechanics of the Incoloy^®^-800H corrosion were not discovered in this experiment.
- The cross section of each sample showed a different degree of degradation or corrosion.
- The experiment showed that, in the transition area, the corrosion is at an intermediate stage. As can be seen in the SS316L cross section, the lower area had highly degraded intergranular corrosion; in the transition area, the intergranular corrosion is clearly seen; and, in the upper area, the intergranular corrosion is shallow and barely visible.
- The cross section of the samples shows the different depth of penetration in each area of different elements into the base metal, as can be seen in Hastelloy^®^G35^®^.
- The experiment clearly shows that Cr plays a critical role in the corrosion process in all three areas (lower, transition and upper), which can be seen in the graph of mass loss vs. %Cr in Figure 4, where Hastelloy^®^G35^®^, with 33%Cr, had the highest mass loss, and SS316L stainless steel, with 17%Cr, had the lowest mass loss.

## Figures and Tables

**Table 1:** Composition of the alloys used in this experiment.

Alloy	Ni wt%	Cr wt%	Fe wt%	Mo wt%	Mn wt%
Incoloy^®^-800H	31.59	20.42	47.99	-	0.8
SS316L Stainless Steel	12	17	65	3	2
Hastelloy^®^-G35^®^	56	33	2	8	0.5

## Data Availability

The data that support the findings of this study are available from the corresponding author upon reasonable request.

## Supplementary Materials

The following supporting information can be downloaded at: https://www.mdpi.com/article/10.3390/ma16072679/s1, Figure S1: The XRD data shows the presence of LiF, KF and NaF in Incoloy-800H, Hastelloy®G35 and SS316L samples.
