# Supplementary material for: Corrosion Behavior of Incoloy®800H, Hastelloy®G35® and 316L Stainless Steel in the Molten Eutectic Fluoride Mixture FLiNaK and Its Vapors
Source: Materials (Basel). 2023 Mar 28;16(7):2679. doi: 10.3390/ma16072679 (PMC10095800; doi:10.3390/ma16072679)
Supplement: Supplementary file 1 [file materials-16-02679-s001.zip › materials-2239060-supplementary.pdf]

materials

Corrosion behavior of Incoloy®800H, Hastelloy®G35® and 316L stainless steel in the molten eutectic fluoride mixture FLiNaK and its vapors

Ambati Ramu *, Viliam Pavlik, Veronika Sillikova, Miroslav Boca

1 *Institute of Inorganic Chemistry, Slovak Academy of Sciences; Dubravska cesta 9, 854 36 Bratislava, Slovakia*
* Ambati Ramu, Email: ramu.ambati@savba.sk

The two examples of XRD pattrens of FLiNaK salt after corrosion experiments are presented showing only the LiF, NaF and KF.

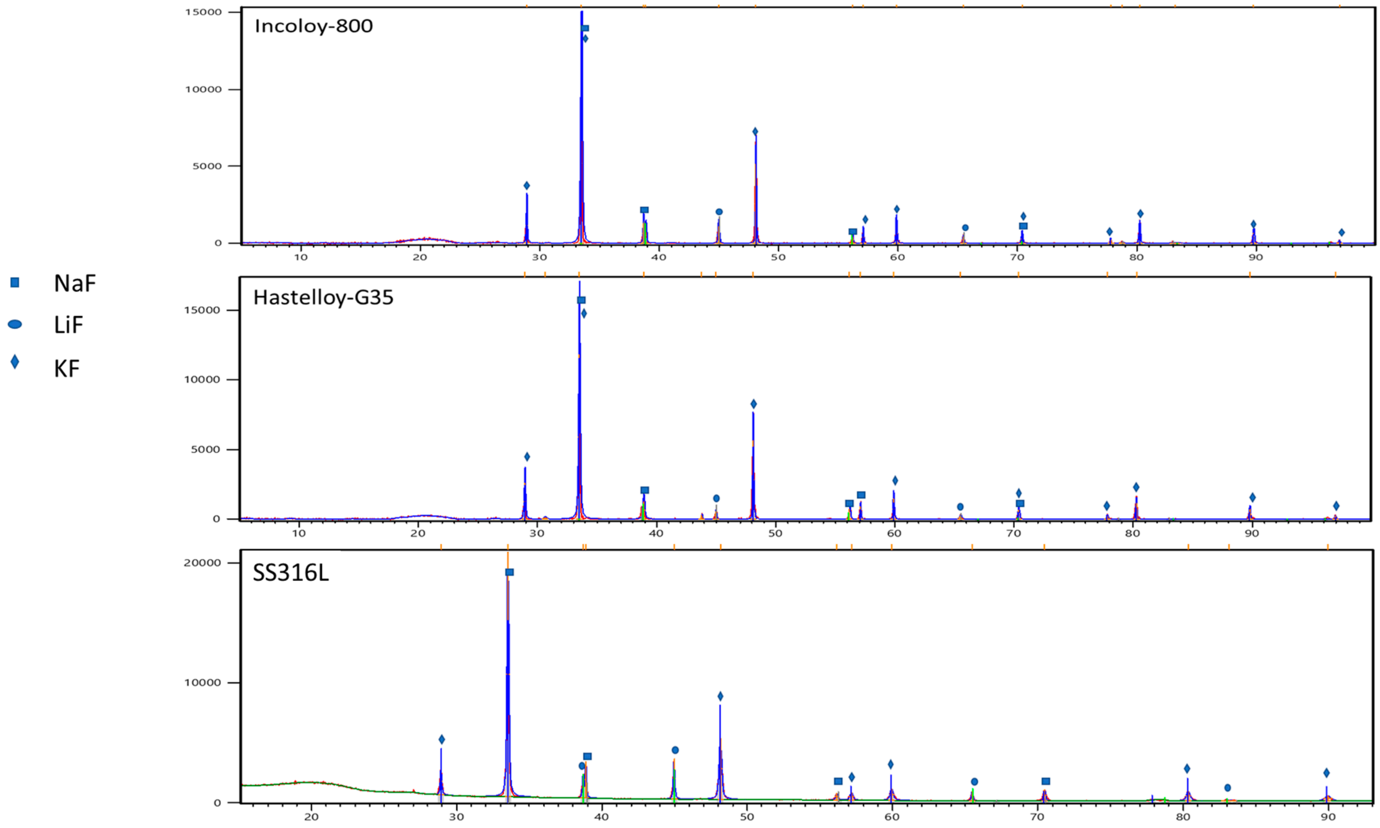

Figure S1 The XRD data shows the presence of LiF, KF and NaF in Incoloy-800H, Hastelloy®G35 and SS316L samples
